# Scheduled telephone visits in the veterans health administration patient-centered medical home

**DOI:** 10.1186/1472-6963-14-145

**Published:** 2014-04-01

**Authors:** Nina R Sperber, Heather A King, Karen Steinhauser, Natalie Ammarell, Susanne Danus, Benjamin J Powers

**Affiliations:** 1 Center for Health Services Research in Primary Care, Durham VAMC, Legacy Tower (NC Mutual Building) Suite 600, 411 West Chapel Hill Street, Durham, NC 27701, USA; 2 Department of Medicine, Division of General Internal Medicine, Duke University, 411 West Chapel Hill Street, Durham, NC 27701, USA; 3 St. Luke’s Health System, 190 E Bannock St, Boise, ID 83712, USA

**Keywords:** Health services research, Telemedicine, Primary care

## Abstract

**Background:**

The Veterans Health Administration (VHA) patient-centered medical home model, Patient Aligned Care Teams (PACT), includes telephone visits to improve care access and efficiency. Scheduled telephone visits can replace in-person care for some focused issues, and more information is needed to understand how this mode can best work for primary care. We conducted a study at the beginning of PACT implementation to elicit stakeholder views on this mode of healthcare delivery, including potential facilitators and barriers.

**Methods:**

We conducted focus groups with primary care patients (n = 3 groups), providers (n = 2 groups) and staff (n = 2 groups). Questions were informed by Donabedian’s framework to evaluate and improve healthcare quality. Content analysis and theme matrix techniques were used to explore themes. Content was assigned a positive or negative valuation to indicate whether it was a facilitator or barrier. PACT principles were used as an organizing framework to present stakeholder responses within the context of the VHA patient-centered medical home program.

**Results:**

Scheduled telephone visits could potentially improve care quality and efficiency, but stakeholders were cautious. Themes were identified relating to the following PACT principles: comprehensiveness, patient-centeredness, and continuity of care. In sum, scheduled telephone visits were viewed as potentially beneficial for routine care not requiring physical examination, and patients and providers suggested using them to evaluate need for in-person care; however, visits would need to be individualized, with patients able to discontinue if not satisfied. Patients and staff asserted that providers would need to be kept in the loop for continuity of care. Additionally, providers and staff emphasized needing protected time for these calls.

**Conclusion:**

These findings inform development of scheduled telephone visits as part of patient-centered medical homes by providing evidence about areas that may be leveraged to most effectively implement this mode of care. Presenting this service as enhanced care, with ability to triage need for in-person clinic visits and consequently provide more frequent contact, may most adequately meet different stakeholder expectations. In this way, scheduled telephone visits may serve as both a substitute for in-person care for certain situations and a supplement to in-person interaction.

## Background

US health systems transformation to patient-centered medical homes (PCMH) includes innovation in primary care delivery, such as the way to access care that is provided by scheduled telephone visits. The PCMH model generally makes use of interdisciplinary teams to communicate with patients in a comprehensive, coordinated and efficient way [1]. Though primary care clinics have long communicated with patients through telephone, usually as a response to patient initiated requests or to follow-up on a specific procedure [2], the telephone and other forms of remote communication are now promoted as alternatives to traditional face-to-face visits, offering patients more flexibility with encounter modes and schedules [3]. Accordingly, more policies are covering telephone-based care, although reimbursement varies by state and may depend on approach, such as integrating video with audio conferencing [4-6].

As in the private sector, scheduled telephone visits have enabled Veteran’s Health Administration (VHA) clinics to expand the range of services and open schedules for same-day/walk-in medical appointments as part of its new PCMH model, known as Patient Aligned Care Teams (PACT). While fee-for-service payment policies may inhibit wider adoption of scheduled telephone visits in the general health care setting, reimbursement for telephone care is not a barrier for implementation within the VHA, the largest integrated health system in the US. Instead, more phone-based encounters are included as one indicator of PACT progress [7-9]. Thus, telephone visits are playing a major role in primary care change toward patient-centered medical homes in the VHA; however, more information is needed from stakeholders to better understand how this mode can most effectively be used for primary care.

Although prior studies have shown that telephone visits can successfully replace in-person care for focused issues, such as follow-up or monitoring [3,10,11], there is little information on how it works for more complex and multifaceted tasks of care coordination, chronic disease management, and preventive health care typically provided in a primary care visit. One study on the impact of substituting telephone care for some VHA clinic visits showed that this mode, with more frequent contact but fewer face-to-face interactions, lowered rates of healthcare utilization with no adverse effects on patient satisfaction [12]. Additionally, researchers have found that primary care users prefer telephone over in-person communication for focused needs such as general medical questions or follow-up [13] and that the telephone can potentially be used for stable, albeit complex, chronic and mental health care [14-16]. The telephone has also generally been successful for increasing uptake of preventive health programs [16]. Scheduled telephone visits thus hold promise as a way to enhance both efficiency of and access to some aspects of patient care. Because the VHA is moving toward more widespread use of scheduled telephone visits, the VHA provides a good setting for further exploring conditions where telephone visits could be used in place of face-to-face primary care encounters [17].

We conducted a study within the VHA at the very beginning of PACT implementation, when scheduled telephone visits were beginning to be promoted by some clinics but had not yet been systematically implemented, to understand potential facilitators and barriers to using scheduled telephone visits as a substitute for in-person visits. An important part of effectively developing new healthcare practices is connecting with potential users to understand what they value and ultimately foster a shared understanding [18], and thus our aim was to assess what patients and primary care team members want from scheduled telephone visits to best meet their needs*.* In a series of focus groups, we asked these stakeholders about their views on structural and procedural aspects of having scheduled telephone visits function as an in-person clinic visit substitute, including who would benefit, what would be the focus, and how would care optimally be structured.

Two frameworks, one theoretical and the other programmatic, were used to help guide data collection and analysis. Constructs from Donabedian’s model to evaluate and improve healthcare quality [19] informed development of focus group questions around theoretical concepts important for evaluating delivery of scheduled telephone services. Because this study was formative, in that it was designed to inform innovation development rather than assess outcomes, we focused on the concepts of structure, which deals with the context in which care is delivered, and process, which deals with transactions between patients and providers during care delivery. Questions concerning structure focused on roles of different providers and characteristics of appropriate patients. Questions concerning processes focused on the exchange between the patients and providers, such as what scheduled telephone visits should cover, and how this encounter might be integrated in the provider’s workflow. Principles guiding the VHA PACT program [7] (see PACT Principles) were additionally used at the point of data analysis to help place findings more specifically within the context of VHA patient centered medical home program priorities.

### PACT Principles*

*Patient-driven*

• The primary care team is focused on the whole person

• Patient preferences guide the care provided to the patient

*Team-based*

• Primary care is delivered by an interdisciplinary team led by a primary care provider facilitative leadership skills.

*Efficient*

• Patients receive the care the need at the time thay need it from an interdisciplinary team functioning at the highest level of their competency.

*Comprehensive*

• Primary care is the point of first contact for a range of medical, behavior, and psychosocial needs, and is fully integrated with other VA health services and community resources.

*Continuous*

• Every patient has an established and continuous relationship with a personal primary care provider.

*Communication*

• The communication between the patient and the other team members is honest, respectful, reliable, and culturally sensitive.

*Coordinated*

• The PACT coordinates care for the patient across and between the health care system including the private sector.

*Reprinted with permission from Klein S. The Veterans Health Administration: Implementing Patient-Centered Medical Homes in the Nation’s Largest Integrated Delivery System. Commonwealth Fund Publication. 2011; 1537:16.

A contribution from this study is the in-depth understanding of how scheduled telephone visits might work for patients and interdisciplinary team members in primary care as a substitute for in-person visits and within the context of a patient-centered medical home model. This research informs development of scheduled telephone visits as part of patient-centered medical homes by providing evidence about areas that may be leveraged to most effectively implement this mode of care. Findings can be used by health services professionals in other settings to help bolster adoption of this health care innovation.

## Methods

### Setting and participants

We conducted focus groups with patients, primary care providers, and staff members at the Durham Veterans Affairs Medical Center (VAMC) and a nearby, community-based outpatient clinic affiliated with the Durham VAMC. Primary care provider groups included physicians, physician assistants, nurse practitioners, advance practice clinical pharmacists, including clinic directors and associate chiefs of staff. The clinic and support staff group included nurses (RNs and LPNs) and nursing assistants as well as administrative support staff who manage the clinic schedule. All providers and staff members in the clinics were eligible for participation. After receiving support from the clinic, these individuals were recruited by email or in-person requests to participate in the study. Patients were selected and recruited based on having a scheduled in-person appointment at the clinic site on the same day of a planned patient focus group. We anticipated that patients most likely to use telephone visits would be those with chronic disease and frequent visits with their providers; thus, inclusion criteria required one or more of the following chronic diseases: hypertension, diabetes, coronary heart disease, congestive heart failure, or COPD; at least 2 VHA primary care visits in the preceding year; and able to communicate regularly over the telephone. Patients were excluded based on: a diagnosis of dementia, cognitive impairment, or psychosis; requirement of regular in-person visits due to clinic injections or anticoagulation monitoring; or being a VHA employee. Patients who met initial inclusion criteria and were still eligible after chart review were sent an introductory letter and then called for screening/scheduling. We selected the first seven patients who responded and were available to attend the planned focus group.

### Data collection and analysis

Focus groups were conducted between August and November of 2010, when VHA primary care was preparing to transform its clinic organization according to principles of the patient-centered medical home, and, at the time of the study, these specific clinics had not yet implemented changes. Though some participants may have been familiar with the PCMH model, the focus of discussions was on individuals’ evaluations of telephone visits as a substitute for some in-person primary care (see Focus group questions for VHA stakeholders regarding scheduled telephone visits). Focus groups were conducted by one researcher, ranged from 4 to 12 participants, and lasted approximately one hour each. We conducted a total of 7 focus groups with patients (n = 3 groups), primary care providers (n = 2 groups), and staff members (n = 2 groups). All focus group discussions were audio-recorded and transcribed; all participants provided written informed consent. The study was approved by the Durham VAMC Institutional Review Board (IRB#1481). Lunch was provided as the only incentive for participation.

### Focus group questions for VHA stakeholders regarding scheduled telephone visits

I want to make a distinction between telephone visits that you do above and beyond your in-person care that supplements the in-person care you receive versus telephone visits that serve as a substitute for some of the things that you currently do in person in your clinic visits. Think about telephone visits as a substitute for some of that in-person care.

How might these telephone contacts be structured within day-to-day delivery of primary care as a means of replacing some in-person encounters? If such telephone visits were scheduled, what would they look like?

How would you want these visits to be incorporated into your day?

Discuss what kind of home monitoring of chronic disease would be necessary for this to work.

Discuss the impact this care would have on the patient-provider relationship.

How would this affect your rapport with your (patients/provider)?

Discuss the impact you think this care would have on the quality of care delivered in primary care.

Discuss the advantages and disadvantages of using the telephone, Internet, or other technologies as the main format for these interactions.

For patients only:

Discus the impact this type of care would have on your satisfaction with your care.

Discuss the impact you think this care would have on the accessibility of primary care for you.

For providers and staff only:

Are there particular groups of patients that may be most appropriate for this type of care? Are there patients who would be inappropriate?

Patients also completed brief written surveys which assessed their transportation and distance to their appointments as well as telephone access. Background information (i.e., age, diagnoses, gender, race) was obtained from a chart review and abstraction of the patients’ electronic medical records as well as from an original data pull of eligible patients. Primary care providers and staff members were surveyed on demographic variables (i.e., age, gender), as well as clinical background (i.e., education/degree, years practicing), and practice characteristics (i.e., current primary care panel size, number of half-day clinic sessions per week).

To analyze qualitative data, we used content analysis and theme matrix techniques [20,21]. Initial coding scheme and matrices were developed by two analysts (NA and BP) and then further refined and developed by two other analysts (NS and HK). The codebook was developed based on focused themes derived from initial coding of the data [22] and *a priori* codes from the Donabedian [19] framework (e.g., characteristics of appropriate patients). Some codes were assigned a positive or negative valuation, depending on whether they reflected facilitators or barriers for implementation of telephone visits. For example, “efficiency” and “satisfaction” were positively valued codes and “unsuccessful” and “uncomfortable” were negatively valued codes. Quotes associated with positive and negatives codes were separately aggregated and displayed in matrices with columns identifying the following dimensions of each quote: characteristics of patients (who), types of situations (what), and means of delivery (how). Matrices were additionally developed to display text coded as relevant for implementation. Separate matrices were developed for each stakeholder category- patients, providers, and staff, and, from these matrices, we identified prominent themes among stakeholder groups. These themes aligned with some VA PACT principles as presented by Klein [7], and thus these principles were used as an organizing framework to present stakeholder reactions within the context of the VA PACT program. Data were managed with Atlas.ti 5.2 [23].

## Results

### Characteristics of participants

The sample consisted of patients (n = 18), primary care providers (n = 16), and staff members (n = 18). All patients were male and most were at least 55 years of age (n = 15; ranging from 42 to 88 years of age, mean = 63.56, standard deviation = 11.38). With regard to chronic health conditions, patients had hypertension (n = 16), diabetes (n = 9), coronary heart disease (n = 7), congestive heart failure (n = 2), and/or chronic obstructive pulmonary disease (n = 2). Most patients had a home telephone (n = 14) or cell phone (n = 14). Over a third of primary care providers were male (n = 6), and most were under 55 years of age (n = 13) and had an MD degree (n = 11). On average, providers had been practicing about 13 years with a current panel size of approximately 599 patients. Over half of providers (56%) reported having 7 to 9 half-day clinic sessions per week. Few staff members were male (n = 3). Most staff members were under the age of 55 (n = 10) and were LPNs degree (n = 8). Additional participant characteristics are provided in Table 1.

**Table 1:** Characteristics of patients, providers, and staff who completed the focus groups*

**Variable**	**Patients (n = 18)**	**Providers (n = 16)**	**Staff (n = 18)**
Male (n (%))	18 (100)	6 (38)	3 (17)
Age (n (%))^†^			
25-34	0 (0)	2 (13)	2 (12)
35-44	1 (6)	6 (40)	4 (24)
45-54	2 (11)	5 (33)	4 (24)
55-64	8 (44)	2 (13)	7 (41)
65+	7 (39)	0 (0)	0 (0)
White (n (%))^†^	8 (53)	-	-
Chronic Health Conditions (n (%))^‡^			
Hypertension	16 (89)	-	-
Diabetes	9 (50)	-	-
Coronary heart disease	7 (39)	-	-
Congestive heart failure	2 (11)	-	-
Chronic obstructive pulmonary disease	2 (11)	-	-
Have a home (land line) telephone (n (%))	14 (78)	-	-
Have a mobile or cell (n (%))	14 (78)	-	-
Drive self to VA appointments (n (%))	14 (78)	-	-
Distance traveled (one-way)			
(Mean (SD)), (Min, Max)	30.7 (17.1), (2–65)	-	-
Degree/license (n (%))			
MD	-	11 (69)	-
PharmD	-	2 (13)	-
PA	-	2 (13)	-
NP	-	1 (6)	-
RN	-	-	5 (28)
LPN	-	-	8 (44)
Other	-	-	5 (28)

*Percentages within a category may not add to 100% due to rounding error.

^†^1 provider and 1 staff member did not respond to the age question. For the patient race question, 1 patient declined to answer and race was unknown for 2 other patients.Participants with missing data were excluded from percentage calculations.

^‡^Participants could have more than one chronic condition diagnosis.

SD = standard deviation.

### Themes relating to VA PACT principles

Themes from focus group discussions with each type of stakeholder were identified relating to the following PACT principles: comprehensiveness, patient-centeredness, and continuity of care (Table 2). Additionally, logistic considerations for implementing scheduled telephone visits were identified by stakeholders (Table 3). Below, we discuss how themes within each of these categories were manifested among groups.

**Table 2:** Potential impact of scheduled telephone visits in the VHA according to PACT principles and stakeholder assessments*

**PACT principles**	**Analytic themes**	**Patients**	**Providers**	**Staff**
Comprehensiveness: Primary care as point of contact for range of patient needs, including mental and physical health	*Using scheduled telephone visits for routine physical and mental health issues*	Routine visits that do not require physical examination (eg, chronic disease monitoring or mental health check-in/follow-up); Determine need for in-person visit	Routine visits that do not require physical examination (eg, chronic disease monitoring); Determine need for in-person visit	Routine visits that do not require physical examination (eg, chronic disease monitoring)
	*Defining scope of scheduled telephone visits- how focused should they be?*	General issues	Focused issues	General issues
Patient-centeredness: Focus on patient wants, needs and preferences	*Engaging patients in determining scheduled telephone visit usage*	For those who choose; want flexibility to change to in-person visits if not comfortable	For those who chose	Can improve patient satisfaction with more patient control over issues discussed
	*Dealing with patients who have adherence or communication challenges*	Could be beneficial for patients anxious about facing provider	For those who are “compliant” and do not have cognitive/verbal difficulties	Concern about liability with higher risk patients (eg, unable to communicate well over the phone)
Continuity: Established and sustained relationship with primary care provider	*Ensuring availability of providers*.	Maintain provider awareness of decisions or subjects discussed via telephone; concern about impersonal aspects of remote encounters	Better for patients with established provider relationships;	Maintain provider awareness of decisions or subjects discussed via telephone and some direct patient contact with providers
	*Strengthening quality of patient-provider relationships*	Better for patients with established provider relationships and can help strengthen relationships with more frequent contact	Can help maintain relationships and improve care quality if patients use preferred mode	

*PACT principles from Klein [10].

**Table 3:** Logistical considerations for implementing scheduled telephone visits by stakeholder category

**Analytic themes**	**Patients**	**Providers**	**Staff**
*Concerns about time- potential time saver, but for whom?*	Less time with reduced travel and briefer visits.	Avoid increase in patient panel size.	Avoid increase in patient panel size.
	Block of time preferable for receiving call.	Provide designated time for calls.	Provide designated time for calls.
		Use staff members to help make calls.	Need more staff support to cover calls.
		Concern about spending time on hard-to-reach patients.	Concern about spending time on hard-to-reach patients.
*Integrating telephone care with other modes of remote communication*.		Tiered system, starting with email and then phone.	Has convenient features, for example surrogate message forwarding, but would not work for every patient.

#### ***Comprehensiveness***

Comprehensive care within PACT means having primary care services as a point of contact for a range of patient needs, including mental health and multimorbid chronic conditions [2,12]. An objective is to efficiently and conveniently help patients by either addressing their various needs or referring them to other VA or community based resources. Along these lines, respondents commented on potential content and scope of scheduled telephone visits, providing some information about the extent to which this mode of care delivery could meet that objective of addressing diverse types of needs within scheduled remote visits.

*Using scheduled telephone visits for routine physical and mental health issues.* Generally, all stakeholder groups said that the telephone would be appropriate for routine kinds of visits that can be done without a physical examination and at home. Examples included monitoring blood sugar or glucose and, from a patient group, mental health check-in/follow-up. Patients and providers suggested that telephone visits could help reduce unnecessary in-person visits by providing an opportunity to evaluate whether an in-person visit would be warranted rather than having it automatically scheduled according to the calendar.

*“Two thirds of the time I’m down here because I got a letter from my care provider…but I feel fairly good and I may not have to see him…If you have a system where you can get through and you can talk, I think you can relieve a lot of this beaurocracy…if he deemed he had to see me then we could go from there.”* -patient

*“Sometimes you can save visits. And if it’s a pretty simple thing, ‘Well, gosh, I didn’t need to see the person anyway. I’ll just postpone it for another two or three or four months or whatever makes sense.”* –provider

*Defining scope of scheduled telephone visits- how focused should they be?.* There was some difference between provider and other stakeholder assessments about whether the telephone would be more appropriate to address general versus focused issues. Patients and staff said that telephone visits could provide an opportunity for patients to ask a range of questions not focused on a single issue. However, in a provider group, it was said that telephone visits work best for more focused problems.

*“…A lot of the time when you do a patient call-back, they will address … most likely another issue, whether it be the paperwork or something else… So even though you called to check on …their blood sugar, they might say, ‘Well and I have to get the doctor to sign this paper”…Even though it might take a little bit longer on the phone call, …they don’t have to call back and say, ‘Oh by the way we were only talking about blood pressure or blood sugar but I had this too.’”* -staff

*”I usually have patients come in and see me and then I assess their competence to follow up over the phone. And I actually find that my phone follow-ups are more focused than my visits. And sometimes there is an ‘Oh by the way’, but I usually just tell them that they need to be seen in clinic and they’re usually fine with that.”* –provider

#### ***Patient-centeredness***

Under PACT, care is to be “patient-driven” with a focus on patients’ wants, needs and preferences [2,12]. This fundamental principle of patient-centered medical homes in essence ensures that various aspects of providing care, from interpersonal to operational level factors, maintain how patients view the world [17]. Data across stakeholder categories underscored this notion of patient-centeredness, revealing that scheduled telephone visits would be highly individualized and depend on personal preferences and patient characteristics.

*Engaging patients in determining scheduled telephone visit usage.* Respondents generally said that it would be important for patients to be involved in determining use of scheduled telephone visits. Staff hypothesized that scheduled telephone visits may offer patients more agency in raising topics to be discussed in the clinical encounter compared to in-person visits, where content may be more provider or staff member driven. Patients and providers noted the benefit of knowing that telephone visits were voluntary, and patients said that they would want to be able to opt out, if not satisfied.

*“A lot of times…their visit is for one thing, but they’re so frustrated over something else that they’re wanting to discuss that and not why they’re here for their visit. And it will cut down on frustration and I think it would improve the patient satisfaction, because…it appears that you care by calling them back and saying, ‘Hey, I just want to touch base with you in between your visit and we just want to see how you are doing.’”* –staff member

*“I want the flexibility to be able to change it if I suddenly decide…”* –patient

*Dealing with patients who have adherence or communication challenges.* Stakeholders offered contrasting views on how scheduled telephone visits would work for patients who have challenges related to adherence or communication. Providers suggested that telephone visits would work best for patients with a history of “compliance” and no verbal or cognitive difficulties, while, in contrast, a patient said that she thought it could be helpful for people like her who find it challenging to follow the doctor’s “orders” and thus face their provider in-person. Additionally, staff expressed concern about liability with providing telephone care to what they regarded as higher risk patients, for example, those who have difficulty communicating.

*“I always have so much guilt by the time I have my appointment. I’m her worst patient. I’ve got to be… I fret over it and I really do have some trouble with it mentally…And if it could be done over the phone, some rascal like me would like that.”* -patient

*“What I chose to do with this particular patient was give him a blood pressure machine, ask him to check it twice a day and to call back within a week to tell me what the blood pressure readings were…I mean, this was a patient that had vested interest in being healthy and no pattern of non-compliance before…I think it…saved not only him having to have that extra hour for a nurse blood pressure check…or even another gap appointment to have it checked. So I think it saved everybody.”* -provider

#### ***Continuity***

Continuity of care refers to patient-provider relationships that are well established and sustained [2,12]. In order to maintain these relationships, it is important to facilitate access to patients’ own providers for both urgent and routine needs [17]. Accordingly, respondents said that effectiveness of telephone visits would depend on provider availability and could have implications for improving relationship quality.

*Ensuring availability of providers*. Patients and staff remarked that it would be important for primary care providers to be made aware of treatment decisions or subjects discussed via telephone visits by nursing or ancillary staff to maintain continuity of patients’ care by their own primary care providers. Patients were concerned about impersonal aspects of remote encounters, for example, potentially not having calls answered, and said that they appreciated having their providers call them to check their status. Staff members echoed this point, saying that, although nurses or other staff may be able to cover some aspects of scheduled telephone visits, patients would still want to have direct contact with their primary care providers at some point.

*“Well in the past,…my provider has been on the ball. He had suggested I go to another clinic for another procedure and two days later he called and said, ‘Did you get that appointment?’…So he does follow up on me. I don’t have anything but good praise…”* -patient

*“Some of the telephone care the patients want from the physician, not from the nurse. And so I just think we need to make sure the physician’s available and willing and has the time in their schedule also to make some of these calls.”* –staff member

*Strengthening quality of patient-provider relationships.* Providers and patients viewed scheduled telephone visits as better for Veterans with established VA provider relationships and potentially beneficial for relationship quality. Providers said that telephone visits could help them to maintain their relationships with patients and improve care quality, because patients could use the mode that would best suit them. Patients said that telephone visits could help to strengthen established relationships by enabling them to have more frequent contact with their care team.

*“… [a Veteran] who’s been with his primary provider for some time- I think it would be a lot easier for him to do that than a new person coming in, because…there’s just too many things that could go wrong with somebody just starting their care under the VA.”* -patient

*“I think it could be beneficial in maintaining the relationship that we have with our patients, also….So in theory it could work; it would give them an opportunity to say, my clinic cares about me…”*- provider

#### ***Logistical considerations for implementation***

In addition to the above themes, participants discussed how scheduled telephone visits might fit into their current schedules and workload (Table 3). One prominent topic for both providers and staff was having time to conduct calls. Providers and staff also considered how scheduled telephone visits might be integrated with secure messaging as way to manage their time.

*Concerns about time- potential time saver, but for whom?* Although patients viewed scheduled telephone visits as a potential time saver, with no travel and briefer visits, staff and providers expressed concern about increased workload without additional help. Providers and staff asserted that they would need dedicated time to make the calls, for example with staff using “administrative time” that they would otherwise use to check patients in or out of the clinic, and providers suggested using staff members to help initiate calls. There was concern by staff about having to spend time tracking down patients; accordingly, patients suggested allocating a block of time during which they could receive the call, because, just as with in-person visits, they might not always be exactly on time for their appointments.

*“…this model has to address the time that we have and right now it’s already over-utilized, with patient care, along with other things, and addressing telephone encounters. So I think in the end it ultimately has to come from ancillary support to make it successful.”* – provider

*“Right now, with the staffing that we have, it does not fit at all. So it would have to definitely be set up with more staff and a blocked out time.”* –staff member

*Integrating telephone care with other remote communication*. Providers and staff also discussed integrating telephone visits with secure messaging as a way to flexibly communicate with patients. One provider suggested a tiered system, starting with email and then phone if warranted. A staff member observed that secure messaging would be limited, because she has found that some patients are not able to use it.

*“The sort of email or secure messaging thing may be the savior of telephone medicine…I can do it more easily on my own time…It may be that if we do more emailing…bumping it up to telephone care if things clinically warrant it and then from there…to an office visit if it seems warranted beyond that…”* –provider

*“Secure messaging looks more and more attractive, because you actually can assign a surrogate. We just had experience with that here recently…but…a certain number of patients who either can’t use it for whatever reason or who are not going to use it, don’t have access to it. So they still need the telephone.”* –staff member

## Discussion

The purpose of this analysis was to understand stakeholder responses to the notion of telephone care as a substitute for in-person visits in order to inform development and implementation; results indicate that they viewed telephone visits as potentially advantageous over in-person visits for certain but not all aspects of primary care. Cases in which telephone could substitute for in-person care included routine disease monitoring for patients who were not high-risk for complications. A perceived advantage was that scheduled telephone visits could strengthen patient-provider relationship by giving patients more agency over their visit. For patients and staff members, this meant more frequent phone calls to check in and allow patients to ask questions, and for providers this meant enabling patients to use their preferred mode. Although stakeholders could anticipate the potential for improved relationships with telephone visits, they had concerns: specifically patients feared the possibility of losing touch with their providers, and providers and staff were cautious about potential for increased work load.

Although patients were open to having auxiliary medical personnel contact them by phone, they expressed concern about not having access to their providers. McKinstry et al. [14] found that patients regarded telephone visits as improving access to providers, however, as in our study, quality of telephone care was regarded by patients and providers as better for those with established relationships. One solution for alleviating this concern could be to assure patients that they could arrange to speak with their provider, over the phone or in-person, if desired. It might be that just knowing that they could have direct contact with providers would help to put them at ease, as expressed in the study by McKinstry and colleagues. Also, scheduled telephone visits could be used in a more selective, targeted way with longer intervals between in-person visits. Wasson et al. [12] found that a similar approach did not compromise patient satisfaction or increase health care utilization, and, in fact, was associated with less costly care. Furthermore, in that study, patient views about scheduled telephone visits improved after the experience. Our findings suggest that VHA patients could accept scheduled telephone visits as a partial substitute for in-person care; because our focus groups were conducted before telephone visits were incorporated into practice, we may also find that views become more favorable as PACT progresses.

Preventing provider and staff burnout in primary care is a major concern [24,25], and, while telephone visits could relieve clinic load and open clinic access [9], providers and staff indicated that they have concerns about potential for increased demands in already tight schedules. Provider concerns about boundaries were evident with remarks about telephone visits as better for more focused issues and not open-ended discussion. Although our findings suggest that some patients may feel more comfortable asking a range of questions over the phone, a study in Scotland demonstrated that consultations conducted via telephone were generally more focused, addressing only a singular patient concern primarily regarding new symptoms, treatment problems, or administrative needs (e.g., prescription refills) [15]. Staff also had concerns about having adequate resources and time to reach patients, anticipating that some would be difficult to reach and require multiple attempts. The notion of a tiered approach, in which remote communication is used as a first-level of contact, was mentioned by all stakeholder groups and could be a way to balance patient desire for keeping providers in the loop and patient and staff need for time management. For some, using secure messaging could be a good first mode of contact.

It is important to consider that stakeholders did not assume that scheduled telephone visits would be appropriate for all primary care needs and patients. Although providers noted that telephone visits might work best for patients who do not have difficulty with following treatments, it is interesting to consider that some patients who have compliance challenges might feel more comfortable communicating with their providers over the phone. Hewitt et al. [10] demonstrated that these kinds of conversations could be successfully done over the phone, though this finding contrasts with other research in which patients reported preferring in-person rather than telephone communication for concerns about new or chronic conditions and instructions about treatments [13]. Additionally, researchers have shown that the telephone can be effective for individuals with mental health needs, reducing some stress associated with face-to-face interaction [16]. It may be hard to predict patient responses to telephone visits, and results from this qualitative study indicate a need for flexibility and not a one-size-fits-all approach. Just as McKinstry et al. [14] found, patient engagement in determining use of telephone visits is important. Patients will need to know that they may refuse telephone care if they do not feel comfortable, though in some cases, for example rural settings where it may take more time for patients to access their facilities, telephone consultation may more consistently be used for initial contact.

These results have some limitations. Because focus groups were conducted only at one VA facility and geographic location, these results may not reflect experiences and viewpoints of VA patients nationally. Additionally, providers in the focus groups were mostly MDs. It may be that mid-level providers have different perspectives; however, we were unable to determine this from our data.

## Conclusion

Use of scheduled telephone visits has the potential to improve access to and quality of VHA primary care in line with PACT principles, but participants were cautious regarding application of this approach. A potential advantage is improved patient-provider relationships, with patients having more frequent contact with their providers, should they chose to include scheduled telephone visits in-between in-person visits. However, patients were concerned about provider availability, and medical team members were concerned about increased work load or scope.

Results from our formative study indicate that telephone visits could be used in a targeted way for routine physical and mental health needs as part of a tiered approach to communicate with patients, with flexibility to accommodate patient preferences for telephone versus in-person modes. Future research could include a survey assessment to evaluate perceived benefits and concerns after telephone visits have been in use for a period of time and whether telephone visits yield differential outcomes for certain kinds of patients, for example, those with difficulty adhering to provider recommendations. It would also be interesting to explore how caregivers might be involved or impacted with scheduled telephone visits, and, though not a prominent theme from our groups, concerns about patient confidentiality in this context [26].

In the shorter term, findings from these focus groups with various stakeholders could be used to help improve buy-in and use of scheduled telephone visits. Presenting this service as enhanced care, with ability to triage need for in-person clinic visits, may most adequately meet different stakeholder expectations. In this way, scheduled telephone visits may serve as both a substitute for in-person care for certain situations and a supplement to in-person interaction.

## Competing interests

The authors declare that they have no competing interests.

## Authors’ contributions

NS contributed to analysis and took lead in drafting the manuscript. HK contributed to analysis and helped to draft the manuscript. KS helped with analytic design and manuscript development. NA contributed to data analysis. SD coordinated data collection activities and IRB requirements. BP conceived of the study, moderated focus groups and participated in analysis. All authors read and approved the final manuscript.

## Pre-publication history

The pre-publication history for this paper can be accessed here:

http://www.biomedcentral.com/1472-6963/14/145/prepub
